# Supplementary figures and images for: Laherradurin Inhibits Colorectal Cancer Cell Growth by Induction of Mitochondrial Dysfunction and Autophagy Induction
Source: Cells. 2024 Oct 3;13(19):1649. doi: 10.3390/cells13191649 (PMC11475353; doi:10.3390/cells13191649)

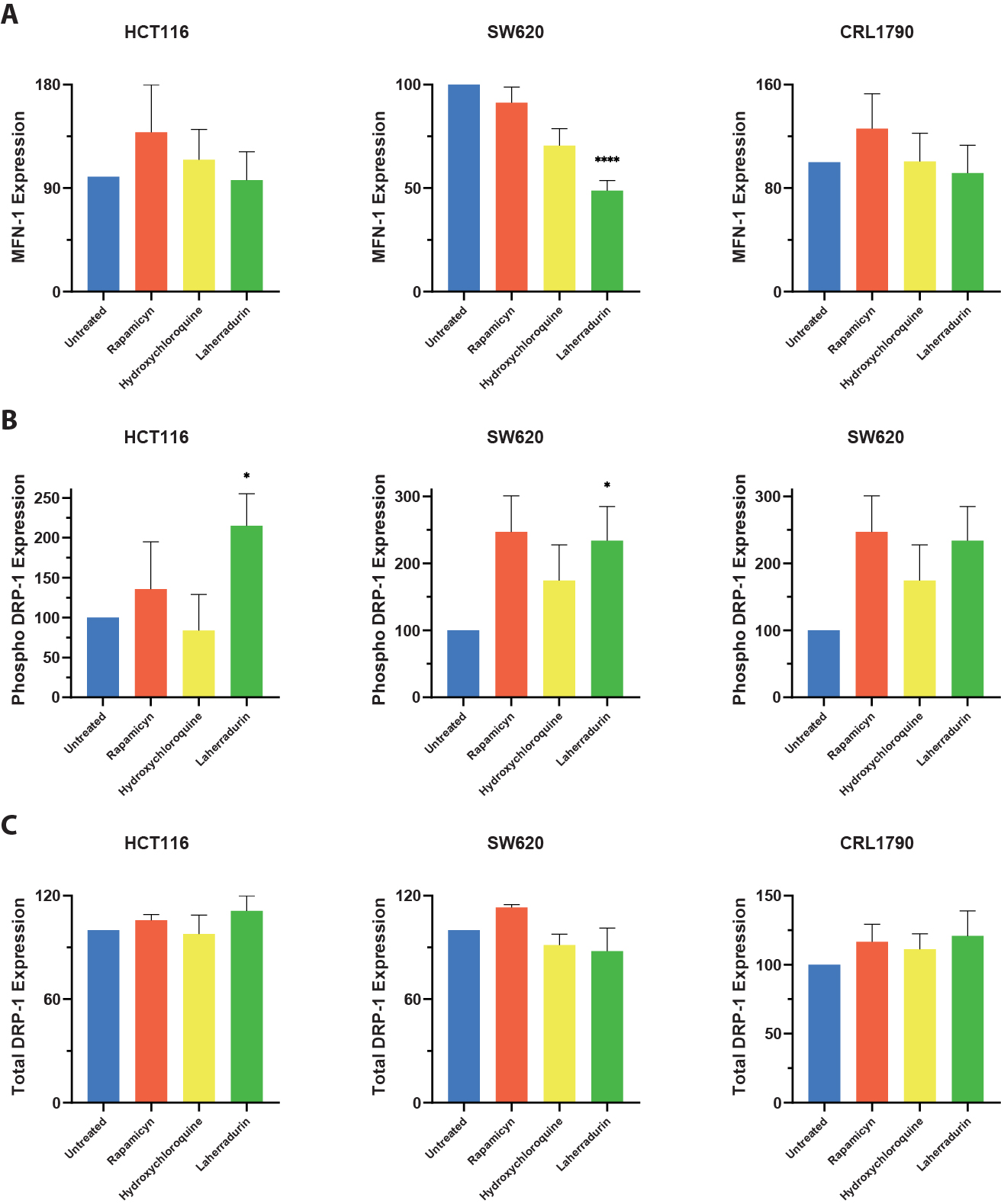

Supplement: Supplementary file 1 [file cells-13-01649-s001.zip › Figure Suplementary 3.jpg]

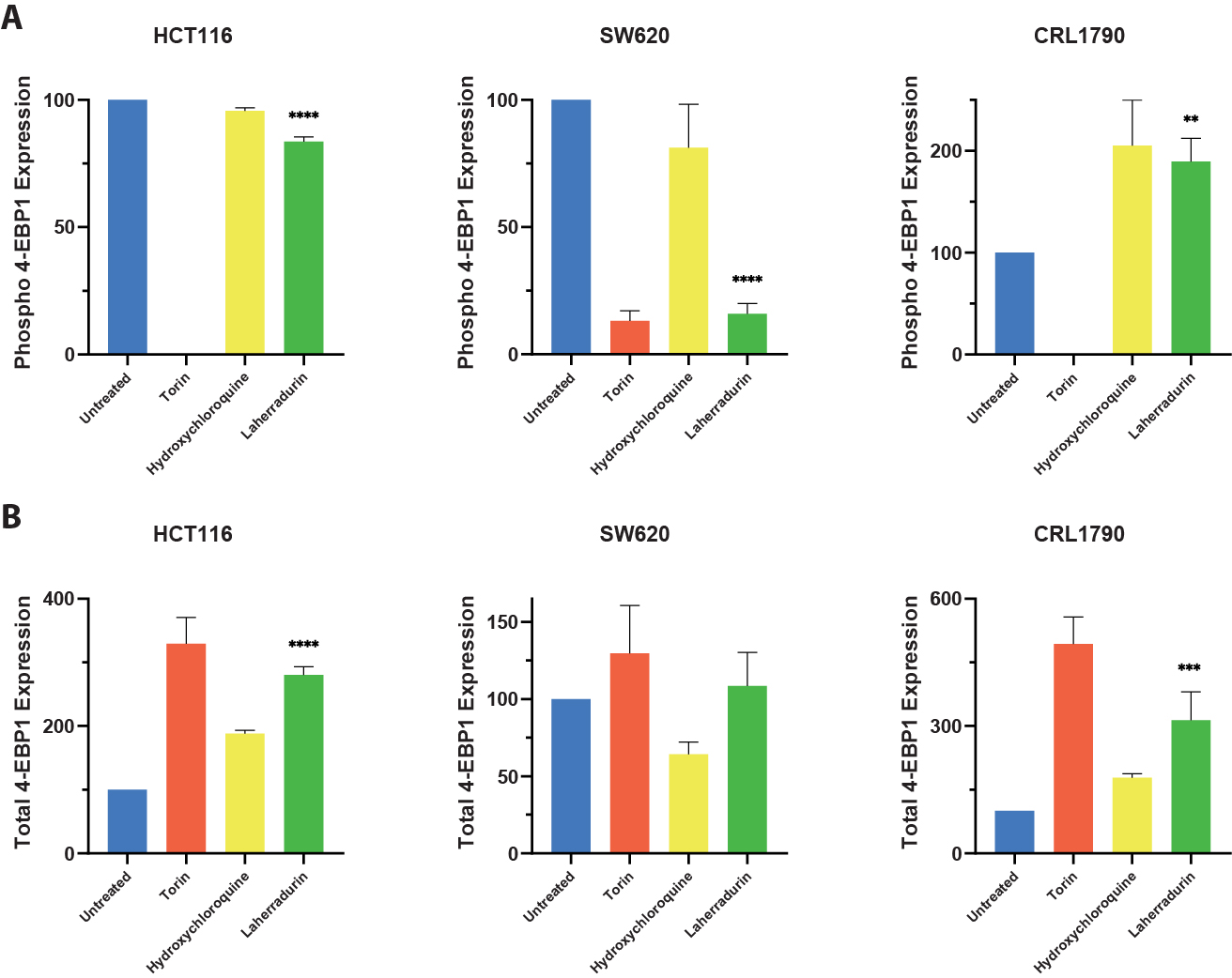

Supplement: Supplementary file 1 [file cells-13-01649-s001.zip › Figure Suplementary 4.jpg]

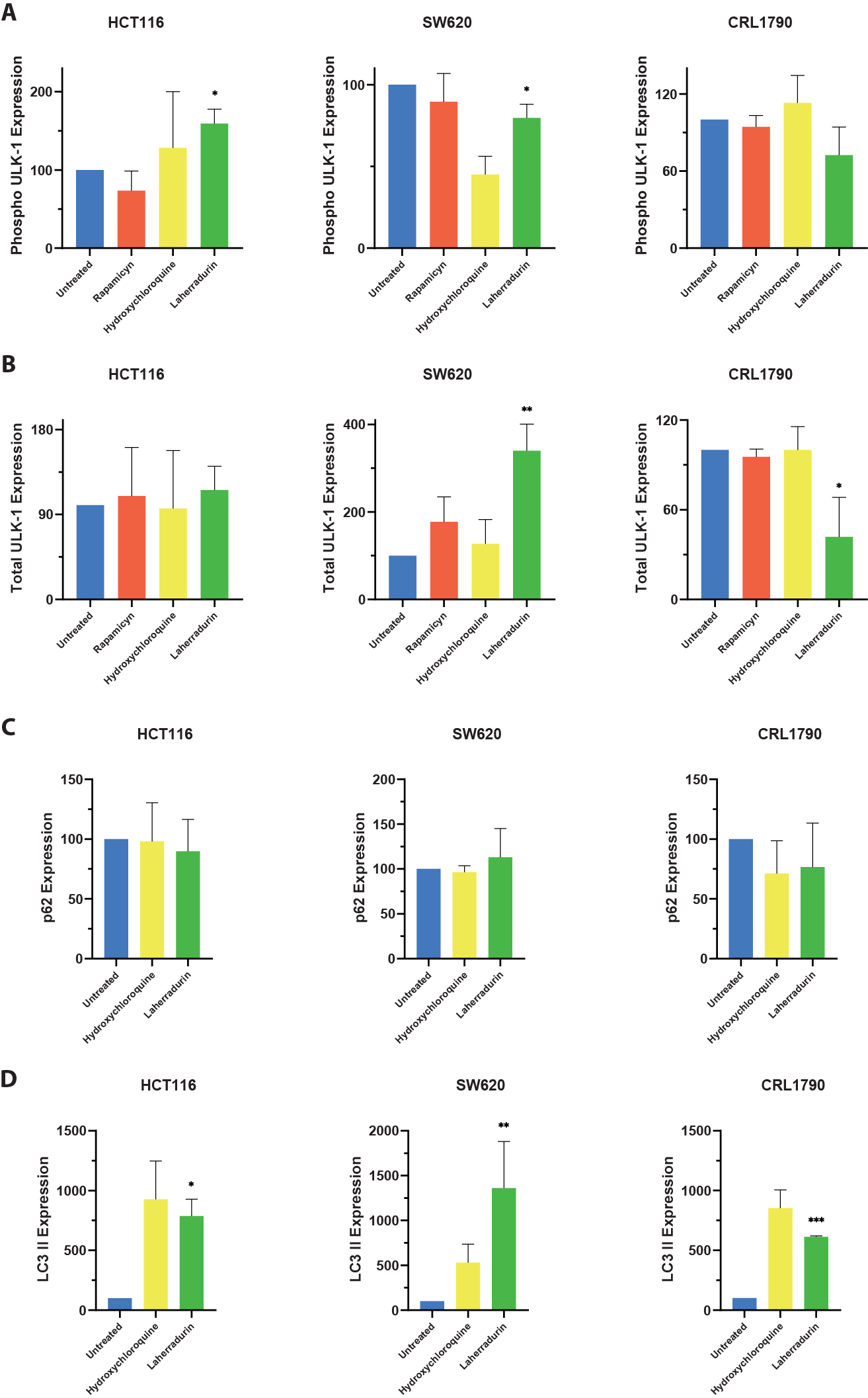

Supplement: Supplementary file 1 [file cells-13-01649-s001.zip › Figure Suplementary 5.jpg]

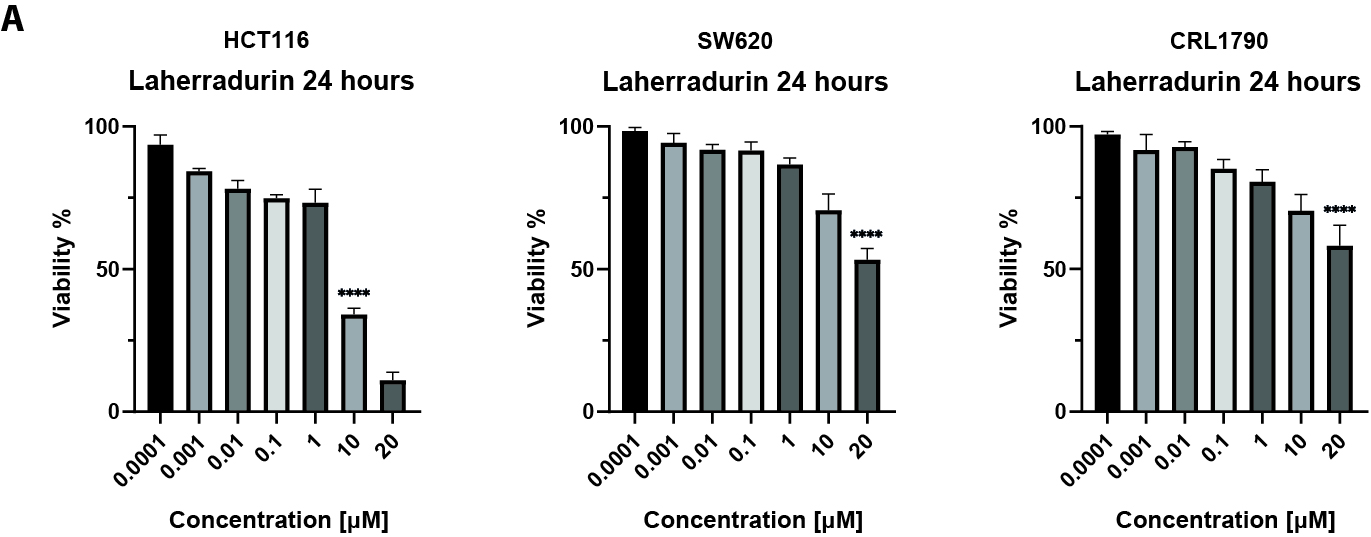

Supplement: Supplementary file 1 [file cells-13-01649-s001.zip › Figure Suplementary 1.jpg]

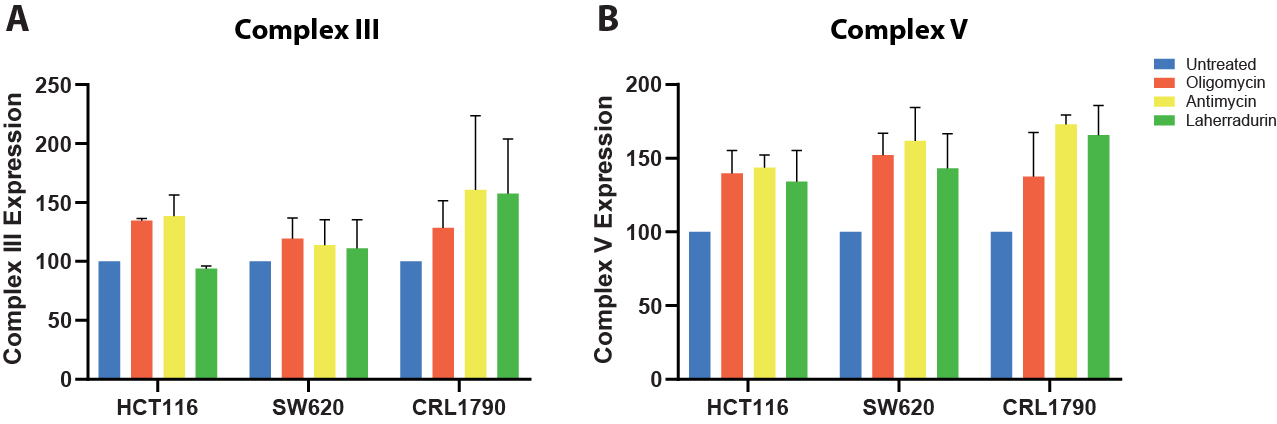

Supplement: Supplementary file 1 [file cells-13-01649-s001.zip › Figure Suplementary 2.jpg]
